# Cardioprotective effects of lycopene-loaded nanoparticles against isoproterenol-induced myocardial injury

**DOI:** 10.22038/ijbms.2025.84316.18241

**Published:** 2025

**Authors:** Xia Liu, Ya Liu, Chunwei Yu, Guang Xu, Xiuyuan Feng, Jia Tang

**Affiliations:** 1 Department of Intensive Care Medicine, The Third People’s Hospital of Chengdu. Chengdu, Sichuan, 610031, China; 2 Department of Cardiovascular Medicine, Ezhou Central Hospital, Ezhou, Hubei, 436000, China; 3 Operating Room, Central Hospital Affiliated to Shandong First Medical University, Jinan, Shandong, 250000, China; 4 Department of Liver and Gallbladder Surgery, Jiangjin District Central Hospital of Chongqing, Chongqing, 402260, China

**Keywords:** Cardiomyoblast Cardioprotective, Chitosan, Lycopene, Myocardial Injury

## Abstract

**Objective(s):**

This study aimed to investigate the cardioprotective effects of lycopene loaded ovalbumin chitosan nanoparticle (L-OCNPs) against isoproterenol (ISO)-induced myocardial injury.

**Materials and Methods:**

H9c2 cardiomyoblasts were treated with various concentrations of L-OCNPs (5, 10, 15, 25, and 50 µg/ml) for 24 hr, followed by exposure to ISO (100 µM) for an additional 24 hr. Cell viability, oxidative stress, mitochondrial function, and nuclear damage were assessed using various biochemical and molecular techniques. Molecular docking studies were conducted to explore the binding interactions between L-OCNPs and the Nrf2 protein.

**Results:**

L-OCNPs exhibited significant cytoprotective effects against ISO-induced cytotoxicity. They effectively reduced oxidative stress by scavenging reactive oxygen species and up-regulating antioxidant enzymes. L-OCNPs also preserved mitochondrial function by maintaining mitochondrial membrane potential and reducing mitochondrial damage. Furthermore, they protected against nuclear damage by inhibiting DNA fragmentation and apoptosis. Molecular docking studies revealed that L-OCNPs, particularly (all-E)-Lycopene and 5Z-Lycopene, interact with the Nrf2 protein, suggesting a potential mechanism of action. Histopathological analysis of rat hearts confirmed the cardioprotective effects of L-OCNPs against ISO-induced myocardial injury.

**Conclusion:**

L-OCNPs demonstrate promising cardioprotective properties by mitigating oxidative stress, preserving mitochondrial function, and preventing nuclear damage. These outcomes propose that L-OCNPs may be a potential therapeutic agent for the prevention and treatment of cardiovascular diseases.

## Introduction

Cardiovascular diseases (CVDs) represent a significant worldwide health concern, including a range of conditions that impact the heart and the complex system of blood vessels (1-3). These disorders are a primary source of global mortality, resulting in millions of deaths each year and highlighting the critical need for a comprehensive knowledge of their intricacies and effective mitigation efforts. The advancement and evolution of CVDs are influenced by a complex interaction of several risk factors, including alterable lifestyle decisions and intrinsic personal traits. Hypertension, dyslipidemia, diabetes mellitus, and tobacco use significantly harm the cardiovascular system (4-7). In addition to these recognized modifiable factors, growing data underscores the significant involvement of obesity, metabolic syndrome, and chronic inflammation in the ethology of CVDs (8-10). Moreover, daily lifestyle choices, such as physical inactivity, unhealthy food practices, and excessive alcohol intake, significantly exacerbate the burden of chronic illnesses, underscoring the need of lifestyle interventions in both prevention and treatment (11).

In cardiovascular research, pharmacological agents such as isoproterenol, a powerful β-adrenergic agonist, are often used to thoroughly examine the processes of heart disease. Isoproterenol has shown efficacy in eliciting myocardial injury in experimental animals, closely resembling certain characteristics seen in ischemic heart disease, a condition marked by diminished blood flow to the cardiac muscle (12-14). Extended exposure to isoproterenol induces a series of harmful biological processes in cardiac myocytes, including an increase in oxidative stress, the activation of inflammatory pathways, and the commencement of apoptosis, or programmed cell death (15). The aggregate effect of this cellular damage results in a substantial reduction in the heart’s capacity to pump blood effectively, ultimately undermining overall cardiac function.

Due to the growing global incidence of CVDs and their significant effect on human health, the creation of novel therapeutic strategies to effectively mitigate and manage myocardial damage has become essential. In the pursuit of innovative therapeutic techniques, naturally occurring chemicals with intrinsic protective qualities have attracted significant interest (16, 17). Lycopene, the vivid red carotenoid pigment prevalent in tomatoes and other red fruits and vegetables, has surfaced as a potential agent for cardio protection (18-20). This natural substance is esteemed for its powerful antioxidant properties, its capacity to neutralize detrimental free radicals, and thus, its ability to alleviate oxidative stress. Previous studies have offered significant evidence of lycopene’s capacity to provide protective advantages in multiple animal models of cardiovascular disease, illustrating its efficacy in diminishing oxidative stress, mitigating inflammation, and preventing apoptosis – all essential mechanisms involved in myocardial damage (21, 22).

Notwithstanding the persuasive cardioprotective potential of lycopene, a considerable obstacle impedes its extensive therapeutic use. The intrinsic lipophilicity of lycopene significantly restricts its solubility in the body’s aqueous milieu. The limited solubility results in constrained absorption in the gastrointestinal system, causing reduced bioavailability, wherein only a little portion of the supplied lycopene reaches the targeted cardiac tissues (23). As a result, its efficacy as a medicinal agent in its original form is significantly limited. The emerging topic of nanotechnology presents a possible option to address the substantial constraint in lycopene delivery. Manipulating materials at the nanoscale facilitates the development of systems that improve medication delivery. Chitosan, a naturally occurring polysaccharide sourced from crustacean shells (24), and ovalbumin, a protein present in egg white (25), have been thoroughly investigated and used as biocompatible and biodegradable carriers for drug administration. Encapsulating lycopene within ovalbumin-chitosan nanoparticles (L-OCNPs) has the potential to markedly enhance its solubility in biological fluids, improve its bioavailability by safeguarding it from degradation and promoting absorption, and allow for targeted delivery to cardiac tissue, thereby optimizing its therapeutic efficacy.

The preparation of these L-OCNPs included a mixing technique to integrate lycopene into the ovalbumin and chitosan matrix. The resultant L-OCNPs were meticulously assessed for their preventive efficacy against isoproterenol (ISO)-induced heart injury. Cell survival, oxidative stress levels, mitochondrial function, and nuclear damage were evaluated using several biochemical and molecular approaches. The results demonstrated that L-OCNPs had considerable cytoprotective properties against ISO-induced cytotoxicity. They successfully reduced oxidative stress by neutralizing reactive oxygen species and enhancing antioxidant enzyme activity. Moreover, L-OCNPs sustained mitochondrial function by preserving mitochondrial membrane potential and mitigating mitochondrial damage, while also safeguarding against nuclear damage by suppressing DNA fragmentation and apoptosis. Molecular docking studies indicated a probable mechanism of action, suggesting that L-OCNPs, namely (all-E)-Lycopene and 5Z-Lycopene, interact with the Nrf2 protein. Histopathological examination of rat hearts further confirmed the cardioprotective properties of L-OCNPs against ISO-induced myocardial damage. Collectively, these findings indicate that L-OCNPs exhibit significant cardioprotective attributes by mitigating oxidative stress, maintaining mitochondrial function, and averting nuclear damage, thereby establishing them as a prospective therapeutic agent for the prevention and treatment of CVDs.

## Materials and Methods

### Chemicals

Cell culture reagents, including Dulbecco’s Modified Eagle’s Medium (DMEM-high glucose), fetal bovine serum (FBS), and trypsin-EDTA, were procured from Gibco-Invitrogen (Carlsbad, CA, USA). The Calcein AM/PI Double Staining kit was obtained from Elabscience (Houston, TX, USA), Rhodamine 123 from Invitrogen (Carlsbad, CA, USA), and the CCK-8 kit, 2’,7’-dichlorofluorescein diacetate (DCFDA), and DAPI from Sigma-Aldrich (St. Louis, MO, USA). All other chemicals and solvents were of analytical grade and prepared fresh before use.

### Preparation of L-OCNPs

L-OCNPs were produced using a simple physical blending technique. The nanoparticles of lycopene (Sigma-Aldrich), ovalbumin (Sigma-Aldrich), and chitosan nanoparticles (NANO^TM^) were mixed together in equal proportions of 1:1:1. The mixture was then mashed using a mortar and pestle to achieve complete and uniform blending. The nanoparticle combination obtained was then placed in a desiccator for further *in vitro* analysis and assessment.

### Cell culture

H9c2 cells were maintained in DMEM supplemented with 10% FBS and antibiotics (streptomycin and penicillin) at 37 ^°^C in 5% CO_2_. The culture medium was changed every 48 hr. Cells were seeded at a density of 5×10^4 ^cells/well in 24-well plates and cultured for 72 hr prior to treatment.

### Cell treatment protocol

The cells were subjected to different doses (5-50 μg/ml) of L-OCNPs. The experimental groups consisted of Control, ISO, 5 μg/ml L-OCNPs+ISO, 15 μg/ml L-OCNPs+ISO, 25 μg/ml L-OCNPs+ISO and 50 μg/ml L-OCNPs+ISO. (I) The control group consisted of cells that were cultured in DMEM for 24 hr and then treated with saline at 37 ^°^C for 24 hr. (II) The ISO group consisted of cells that were cultured in DMEM for 24 hr and then treated with 10 μM ISO at 37 ^°^C for one day. (III) Different concentration of L-OCNPs (5, 10, 15, 25, and 50 μg/ml) groups consisted of cells that were pre-incubated at 37 ^°^C for one day and then treated with 10 μM ISO at 37 ^°^C for one day.

### Cell counting Kit-8 (CCK-8) assay

Cell viability was evaluated via the CCK-8 test. H9c2 cells were cultured in 96-well plates and incubated for 24 hr. Following treatment with L-OCNPs and ISO, CCK-8 reagent was introduced to each well and incubated for four hours. Cell viability was assessed by measuring absorbance at 450 nm using a microplate reader (Mindray MR-96).

### Cell apoptosis analysis

The Calcein AM/PI double staining assay was used to assess apoptosis in H9c2 cells. After 24 hr of culture, cells were stained with Calcein AM/PI and examined under a fluorescence microscope (Olympus-CKX53) to differentiate between live, apoptotic, and necrotic cells.

### Detection of reactive oxygen species (ROS) production

The study measured intracellular reactive oxygen species (ROS) using a fluorescent probe, 2’,7’-dichlorofluorescein diacetate (DCFH-DA). Cells were incubated with 50 µM DCFH-DA for 30 min, and ROS-induced fluorescence was detected using a fluorescent microscope (Olympus-CKX53).

### Antioxidant estimation assay

Following treatment, H9c2 cells were collected and disposed in accordance with the guidelines provided by the SOD, CAT and MDA commercial kits protocol. The absorbance was quantified using an Automated Microplate Reader Mindray MR-96 (Mindray, Nanshan, China).

### Mitochondrial membrane potential staining

Mitochondrial morphology was assessed using Rhodamine 123, a cationic dye that accumulates in mitochondria based on membrane potential. After a 20-minute incubation, cells were washed with PBS and examined under a fluorescent microscope (Olympus-CKX53).

### Nuclear staining by DAPI

Nuclear morphology was evaluated by staining with DAPI. After ten minutes of fixing in 3.7% formaldehyde, the cells were permeabilized for 7 min using 0.3% Triton X-100. Following PBS washing, cells were incubated in the dark for 7 min with DAPI (5 µg/ml). A fluorescent microscope (Olympus-CKX53) was used to visualize nuclear morphology after a final wash with PBS.

### In silico docking analysis

A molecular docking simulation was conducted to investigate the potential interaction between lycopene and Kelch-like ECH-associated protein 1 (KEAP1), a vital component in the Nrf2 signalling cascade. From the PubChem database (https://pubchem.ncbi.nlm.nih.gov/), the 3D structures of 5Z-lycopene (PubChem CID: 11756979) and all-E-lycopene (PubChem CID: 131954643) were attained. The structures were docked prepared by tools such as AutoDockTools, which defined rotatable bonds and assigned suitable atom types for precise interaction prediction. Utilizing the Protein Data Bank (PDB), essential protein structures linked to cardiovascular disease (CVD) were found. KEAP1 (PDB ID: 2FLU), a part of the Kelch-Neh2 complex that controls Nrf2 activation, was the particular subject of our attention. Using AutoDockTools, the retrieved KEAP1 structure was also prepared for docking.

### In vivo trial

The Animal Experimentation Ethics Committee of the Department of Liver and Gallbladder Surgery at Jiangjin District Central Hospital in Chongqing authorized all operations (NO. 2024-19–X28). The current research adhered to the guidelines for the care of laboratory animals. Forty-eight male Sprague-Dawley (SD) rats, weighing between 150 and 230 grams, were randomly allocated into eight groups, each including six rats. The control group was given two subcutaneous injections of normal saline (1.5 ml/kg) on days twenty-seven and twenty-eight. The ISO group was administered two subcutaneous injections of isoproterenol (ISO, 80 mg/kg) on days twenty-seven and twenty-eight to induce myocardial infarction (MI). The six remaining groups were administered varying doses of L-OCNPs (5, 10, 15, 25, and 50 µg/ml) orally for twenty-eight days, succeeded by two subcutaneous injections of ISO (85 µg/ml) on days twenty-seven and twenty-eight. Upon completion of the experiment, the hearts were removed, preserved in 10% formalin, prepared for histological analysis, and marked with hematoxylin and eosin (H&E) for microscopic evaluation.

### Statistical analysis

To determine statistically significant differences among groups, one-way ANOVA was followed by Sidak’s various contrasts trial, a rigorous *post hoc* analysis. Statistical scrutinizes were accomplished using Prism (GraphPad Software, CA, USA). Data are existing as mean ± standard deviation. Statistical implication was set at the highly significant level of *P*<0.0001.

## Results

### Cytotoxicity assessment of L-OCNPs

**Figure 1:**
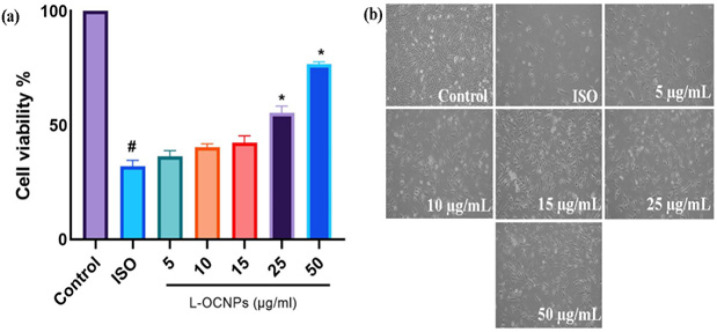
Effect of lycopene loaded ovalbumin chitosan nanoparticle (L-OCNPs) treated against isoproterenol (ISO) in cardiomyoblasts by cell counting kit-8 (CCK-8) assay

The MTT experiment demonstrated that L-OCNPs had exceptional biocompatibility at concentrations up to 25 μg/ml (Figure 1). The experiment exhibited a cell viability rate of about 80%. This suggests that the H9c2 cardiomyoblasts responded well to the nanoparticles and did not observe any significant adverse effects at the given doses. However, as the concentration reached 50 μg/ml, there was a noticeable decrease in cell viability, indicating the initiation of cytotoxic effects. The finding is further supported by the IC_50 _value, which is determined to be 25 μg/ml. It identifies the specific concentration at which L-OCNPs significantly impede cell development. Microscopic studies (Figure 1b) showing a healthy morphology of H9c2 cells exposed to L-OCNPs at non-toxic levels confirmed these results even further. The findings are promising as they suggest that L-OCNPs have potential for biological applications without presenting substantial dangers to cardiomyocytes. The observed biocompatibility may be ascribed to many reasons, such as the inherent biocompatible properties of the individual constituents (lycopene, ovalbumin, and chitosan) and the meticulous manufacturing of the nanoparticles.

### Determinations of apoptosis by Calcein AM/PI Double staining

**Figure 2:**
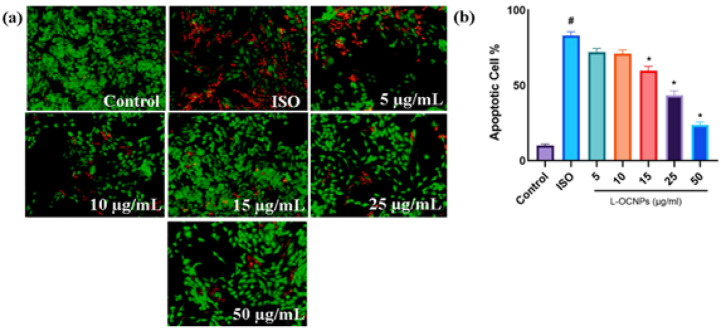
(a) Calcein AM/PI staining of H9c2/isoproterenol treated with lycopene loaded ovalbumin chitosan nanoparticle (L-OCNPs) at different concentrations (5, 10, 15, 25, and 50 µg/ml), AM: green signal, PI: red signal. (b) Bar diagrams represent the apoptotic cells of different concentration of L-OCNPs (5, 10, 15, 25, and 50 µg/ml)

In order to conduct a more thorough examination of the impact of L-OCNPs on H9c2 cardiomyoblasts, the process of Calcein AM/PI staining was carried out (Figure 2a). This method enables the distinction between normal, early apoptotic, late apoptotic, and necrotic cells by assessing their membrane integrity and intracellular activities. H9c2 cells were exposed to L-OCNPs for a duration of one day. Afterward, the H9c2 cells were stained and observed under fluorescent microscope. Cells treated with L-OCNPs at doses of 25 and 50 μg/ml did not show any significant of cell death. Nevertheless, there was a noticeable rise in the number of necrotic cells, which was directly related to the concentration, as seen by their inconsistent orange-red fluorescence. These data indicate that while L-OCNPs do not cause programmed cell death at the dosages examined, they may cause cell death by necrosis, especially at higher concentrations (Figure 2b).

### ROS formation in cardiomyocytes

**Figure 3:**
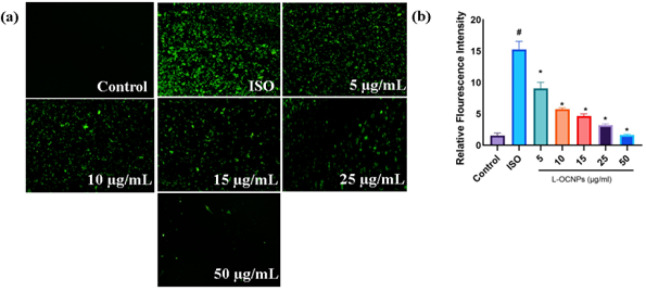
(a) Fluorescence images of reactive oxygen species (ROS) induced by lycopene loaded ovalbumin chitosan nanoparticle (L-OCNPs) at different concentrations, (b) The intensity of ROS fluorescence was calculated using Image J software

In order to examine the antioxidant characteristics of L-OCNPs, we evaluated the production of ROS in H9c2 cardiomyocytes by employing DCFH-DA staining. Figure 3 (a-b) illustrates that isoproterenol therapy markedly increased intracellular ROS levels in comparison to the control group. Significantly, administering L-OCNPs before exposing the cells to isoproterenol led to a substantial decrease in the making of ROS. The results indicate that 25 and 50 μg/ml L-OCNPs have strong antioxidant properties and efficiently reduce oxidative stress caused by isoproterenol. These findings suggest that L-OCNPs possess strong antioxidant properties, making them promising candidates for combating oxidative stress associated CVD.

### Effect of L-OCNPs on antioxidant enzymes and lipid peroxidation

**Figure 4:**
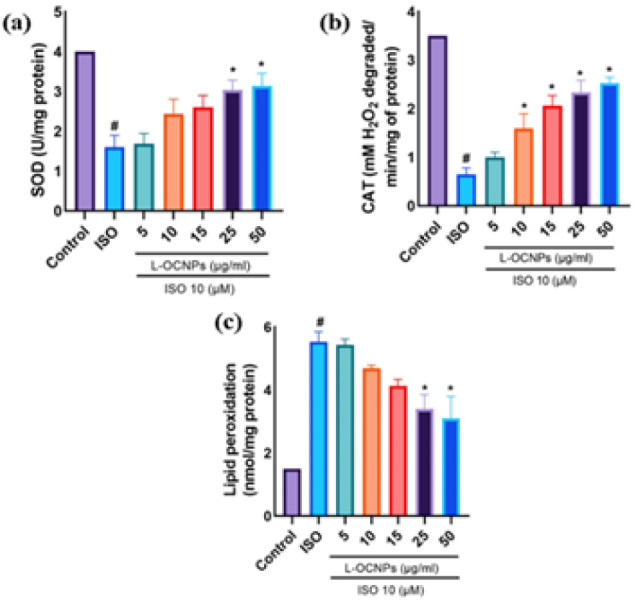
Effect of lycopene loaded ovalbumin chitosan nanoparticle (L-OCNPs) treated against isoproterenol (ISO) injury in antioxidant enzymatic assay catalase, SOP and lipid peroxidation

In order to examine the impact of L-OCNPs on the antioxidant defence system, evaluated the levels of CAT and SOD activity. The administration of isoproterenol resulted in a considerable reduction in the activity of both SOD and CAT when compared to the control group. This suggests that the antioxidant defence system is impaired due to oxidative stress. On the other hand, the administration of 25 and 50 μg/ml of L-OCNPs resulted in a considerable increase in the activities of SOD and CAT compared to the group exposed to isoproterenol (Figure 4a-b). This indicates that L-OCNPs efficiently reduce oxidative stress by improving the mechanism of antioxidant defence. The assessment of lipid peroxidation, which is a characteristic feature of oxidative damage, was conducted by quantifying the levels of malondialdehyde (MDA). The administration of isoproterenol resulted in a substantial rise in MDA levels contrast to the control, indicating an elevation in lipid peroxidation. On the other hand, the administration of L-OCNP resulted in a considerable reduction in MDA planes when comparability to ISO group (Figure 4c). This indicates that L-OCNPs are effective in preventing lipid peroxidation and maintaining cellular membranes against oxidative damage.

### Mitochondrial membrane potential by fluorescence microscopy

**Figure 5:**
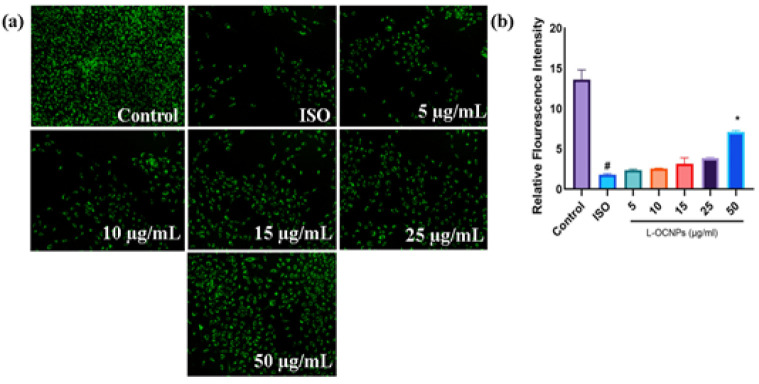
(a) Mitochondrial membrane potential (MMP) in H9c2 cells treated lycopene loaded ovalbumin chitosan nanoparticle (L-OCNPs) at different concentrations (5, 10, 15, 25, and 50 µg/ml); (b) The intensity of reactive oxygen species (ROS) fluorescence was calculated using Image J software

To further understand the processes behind L-OCNPs’ cardioprotective benefits, consider their influence on mitochondrial activity. Mitochondrial dysfunction, defined as an impairment of mitochondrial membrane potential (MMP), is an indicator of cardiac injury. As indicated in Figure 5a-b, isoproterenol administration had significant effect on MMP in H9c2 cardiomyocytes when compared to the control group. Pretreatment with L-OCNPs, especially at dosages of 25 and 50 μg/ml, effectively minimized the reduction of MMP caused by isoproterenol. These data indicate that L-OCNPs maintain mitochondrial integrity and function, reducing myocardial damage. L-OCNPs’ ability to preserve mitochondrial function is most likely owing to their antioxidant characteristics, which aid to minimize oxidative stress and protect mitochondrial components.

### DAPI staining

**Figure 6:**
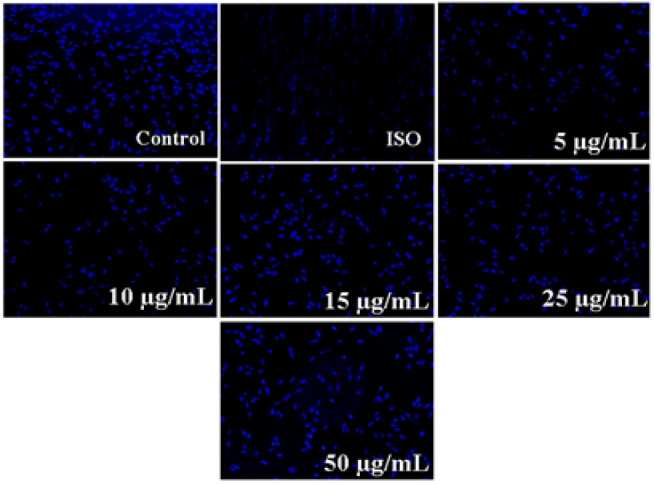
DAPI staining assay showing apoptotic cells with membrane blebbing and apoptotic bodies

To evaluate the effect of L-OCNPs on nuclear integrity, DAPI staining was used (Figure 6). This method enables the observation of nuclear structure and chromatin compaction, which are characteristic of apoptosis. H9c2 cardiomyocytes, when exposed to isoproterenol, displayed distinct apoptotic morphological alterations, such as a decrease in nuclear size and condensation of chromatin. Nevertheless, the administration of L-OCNPs before treatment, especially at dosages of 25 and 50 μg/ml, effectively reduced these nuclear changes. The results indicate that L-OCNPs provide protection against DNA damage and apoptosis caused by isoproterenol. The antioxidant capabilities of L-OCNPs may contribute to the preservation of nuclear integrity by preventing oxidative damage to DNA. 

### Molecular docking analysis of different lycopene isomers to Nrf2

**Figure 7:**
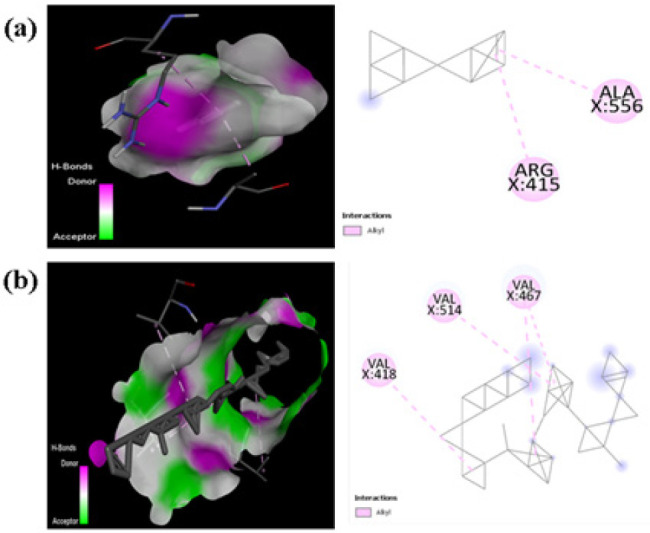
(a) 3D and 2D Interaction for Keap1and 5Z Lycopene, and (b) All–(E-) Lycopene

The study used molecular docking techniques to examine the binding affinities of (all-E)-Lycopene and 5Z-Lycopene in the hypothetical active transporter pocket. The findings demonstrated unique binding orientations and interactions for each isomer, indicating that certain amino acid residues are essential in recognizing and binding to the substrate. 5Z-Lycopene interacted with ARG415 and ALA556 of Keap1, while (all-E)-Lycopene had higher binding affinities for VAL418, VAL467, and VAL514 residues (Figure 7 a-b). The predicted maximal binding affinity between (all-E)-Lycopene and Keap1 protein was determined to be 15.5 kcal/mol (Table 1). These variable interactions are likely responsible for the observed variations in substrate binding and subsequent cellular responses. The unique ways in which (all-E)-Lycopene and 5Z-Lycopene bond inside the transporter pocket may affect how they are absorbed, transported, and made available for use in the body.

### Histopathological analysis

**Figure 8:**
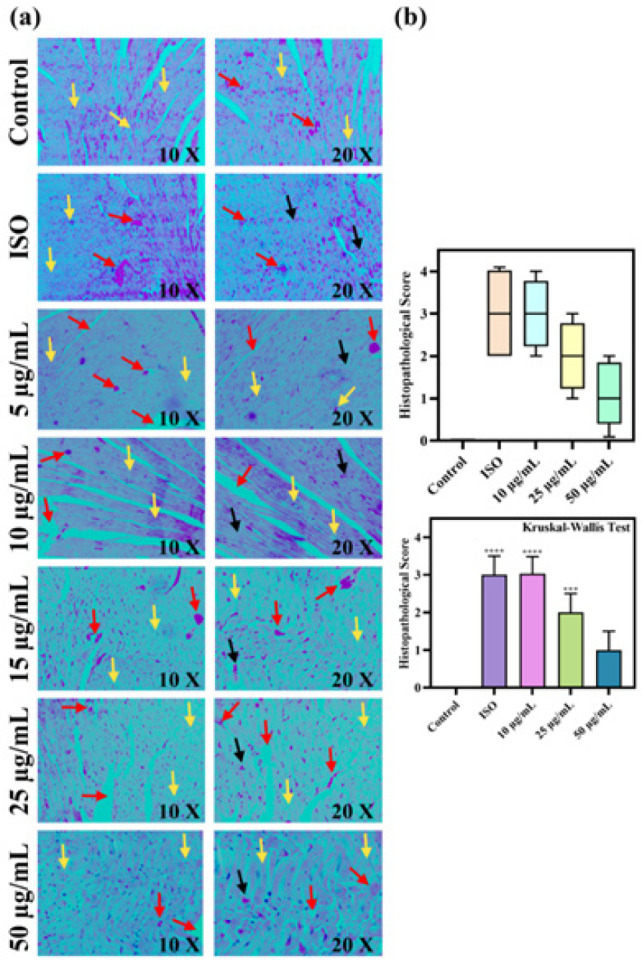
Histopathological analysis of myocardial injury (MI)

Histopathological analysis revealed varied patterns of myocardial injury (MI) across the experimental groups (Figure 8). In the control group, low magnification reveals compact, evenly distributed cardiac fibres (red arrows), whereas high magnification indicates maintained homogenous nuclei and cytoplasmic irritated pattern. In ISO group: low magnification demonstrations an extensive parting of myocardial fibres (MF) owing to interstitial edema (Red arrows), parts of MF lose eosinophilic mark and become whiteness, and inflammatory cells influx into MF. High magnification demonstrations pathological transformations of MI, interstitial edema between MF, and loss of cytoplasmic pattern. In the L-OCNPs (5-10 µg/ml)+ISO group, low magnification shows no significant difference in myocardial fiber separation due to interstitial edema (Red arrows) and inflammatory cell influx (Yellow arrows). High magnification reveals persistent pathological changes of MI, including cytoplasmic striation loss and nuclei shrinkage. In the L-OCNPs (15-25 µg/ml)+ISO group, low magnification shows interstitial edema (black arrows) and a slight reduction in inflammatory cell influx (red arrow). High magnification shows milder pathological changes of MI, including interstitial edema between myocardial fibers, loss of cytoplasmic striation, shrinkage of nuclei, and prominence of the cell membrane. The L-OCNPs (50 µg/ml)+ISO group exhibits compact, uniform myocardial fibers, no interstitial edema, few chronic inflammatory cells, cytoplasm, eosinophilic, few macrophages and even nuclei at low magnification (Red arrows), and few macrophages at high magnification (Yellow arrows). 

## Discussion

This work successfully demonstrates the protective properties of L-OCNPs against isoproterenol-induced injury in H9c2 cardiomyocytes, underscoring their potential as a therapeutic agent for CVDs.

Our preliminary evaluation of biocompatibility indicated that L-OCNPs have exceptional compatibility with cardiomyocytes, exhibiting no negative responses or cellular death at doses up to 50 μg/ml. This fundamental discovery confirms the safety profile of L-OCNPs within the examined parameters, allowing future exploration of their protective mechanisms.

A crucial element of isoproterenol-induced heart damage is the amplification of oxidative stress via heightened reactive oxygen species (ROS) production (26). Our findings clearly illustrate the strong antioxidant capabilities of L-OCNPs, as shown by their substantial decrease in isoproterenol-induced ROS generation. This discovery is further corroborated by the noted increase of essential antioxidant enzymes, catalase and superoxide dismutase (SOD). These results correspond with prior research emphasizing the antioxidant efficacy of lycopene in alleviating oxidative stress and inflammation in hypercholesterolemic animal models (27), indicating a preserved protective mechanism.

Mitochondrial dysfunction is a pivotal factor in myocardial injury (28). The capability of L-OCNPs to safeguard MMP highlights their involvement in sustaining mitochondrial integrity, an essential function given mitochondria’s position as the principal generator of intracellular ROS. Impaired mitochondrial activity may trigger a harmful loop of oxidative stress and cell death. Our results align with previous studies demonstrating that lycopene’s protective effects against cardiac ischemia-reperfusion damage are associated with the inhibition of mitochondrial permeability transition pore opening, indicating a possible shared mechanism of cardio protection.

DAPI staining demonstrated that L-OCNPs significantly reduce nuclear damage, highlighting their protective function against DNA damage and apoptosis. This observation is notably important as nuclear damage signifies a frequently irreversible indicator of cellular injury (29-32). The ability of L-OCNPs to preserve nuclear integrity enhances their potential as a cardioprotective agent.

The convergence of L-OCNPs in reducing oxidative stress, maintaining mitochondrial function, and averting nuclear damage clearly indicates their potential as a viable treatment approach for CVDs. Our molecular docking investigations provide new insights into the varying binding affinities of lycopene isomers to the Nrf2 protein. The unique amino acid interactions identified between (all-E)-Lycopene (VAL418, VAL467, VAL514) and 5Z-Lycopene (ARG415, ALA556) at the Nrf2 interface imply possibly different mechanisms of action for these isomers in regulating Nrf2 activity. These interactions may affect Nrf2 stability or degradation, thereby influencing its downstream antioxidant and cytoprotective functions. These results align with other observations emphasizing the diverse biological actions of lycopene isomers (33, 34).

Histopathological examination provided robust data supporting the cardioprotective effects of L-OCNPs in ISO-induced myocardial damage. Our results corroborate the current literature demonstrating the beneficial benefits of lycopene in diverse CVDs. The differing levels of protection shown in the L-OCNPs-treated groups indicate a distinct dose-dependent impact. Lower dosages (5-10 µg/ml) provided little protection, but a higher concentration (50 µg/ml) demonstrated substantial cardioprotective advantages. This dose-response relationship highlights the need of adjusting L-OCNPs dosage to get optimal therapeutic effectiveness.

The cardioprotective mechanisms of L-OCNPs are likely complex, perhaps including a blend of antioxidant, anti-inflammatory, and anti-apoptotic effects, as shown by other studies (35-38). Our results highlight the potential of L-OCNPs as a potentially therapeutic agent for the prevention and treatment of myocardial damage. Additional study is necessary to clarify the specific molecular pathways responsible for these protective benefits and to enhance their translational potential.

(a) Effect of different concentration of L-OCNPs (5, 10, 15, 25, and 50 µg/ml) on ISO treated H9c2 cells. (b) Microcopic images of different concentration of L-OCNPs (5, 10, 15, 25, and 50 µg/ml). Data are stated as mean±standard deviation in three trials. ^##^*P<*0.0001 vs control. **P<0.0001 vs ISO treated groups

Data are stated as mean±standard deviation in three trials. ^##^*P<*0.0001 vs control. **P<0.0001 vs ISO treated groups

Data are stated as mean±standard deviation in three trials. ^##^*P<*0.0001 vs control. **P<0.0001 vs ISO treated groups

Data are stated as mean±standard deviation in three trials. ^##^*P<*0.0001 vs control. **P<0.0001 vs ISO treated groups

Data are stated as mean±standard deviation in three trials. ^##^*P<*0.0001 vs control. **P<0.0001 vs ISO treated groups

**Table 1:** The affinity of isomers of lycopene and Keap1 protein. The binding affinities of the most suitable binding modes are provided in units of kcal/mol, while the distance from the optimum mode is indicated by the subordinate bound (rmsd l.b.) and superior bound (rmsd u.b.) values

Compound	Binding Affinity (kcal/mol)	Distance from rmsd l.b.	Distance from rmsd u.b.
5Z Lycopene	-14.8	0.000	0.000
All-(E-) Lycopene	-15.5	0.000	0.000

(A) Representative H&E-stained sections of heart tissue from different treatment groups. (B) Histopathological score in tentative animals pretreated with lycopene loaded ovalbumin chitosan nanoparticle (L-OCNPs)(5-50 µg/ml) in an isoproterenol (ISO)-induced MI model. Data are presented as mean±SEM (n=6). Statistical implication was*P<0.005, **P<0.001, ***P<0.0001 associated to the control sample

## Conclusion

This study investigated the potential cardioprotective effects of lycopene-loaded ovalbumin chitosan nanoparticles (L-OCNPs) against isoproterenol-induced cardiac damage in H9c2 cardiomyoblast. L-OCNPs exhibited no significant cytotoxicity at concentrations up to 25 μg/ml, indicating their safety for cardiomyocytes. L-OCNPs effectively reduced intracellular ROS generation and enhanced the movement of CAT and SOD in isoproterenol-treated cells, mitigating oxidative stress. L-OCNPs maintained mitochondrial membrane potential (MMP), suggesting their ability to protect mitochondrial integrity and function, a crucial factor in preventing cell death. L-OCNPs protected against DNA damage and apoptosis as evidenced by DAPI staining, highlighting their potential to prevent irreversible cellular injury. Additionally, the *in silico* docking analysis revealed distinct binding affinities of lycopene isomers with the Keap1 protein, suggesting potential differences in their modes of action. The results provide valuable insights into the mechanisms underlying L-OCNPs’ cardioprotective effects. In conclusion, this study suggests that L-OCNPs hold significant promise as a novel therapeutic strategy for mitigating cardiovascular damage associated with oxidative stress. Future studies should explore their efficacy *in vivo* models and optimize their delivery systems for clinical translation.

## Declaration

We have not used any AI tools or technologies to prepare this manuscript.

## References

[1] Chen X, Xie L, Wu W (2023). Simvastatin reduces high uric acid-induced oxidative stress and inflammatory response in vascular endothelial cells via nuclear factor E2-related factor 2 (Nrf2) signaling. Iran J Basic Med Sci.

[2] Sharma S, Iqubal A, Khan V, Sharma K, Najmi AK, Haque SE (2023). Icariin ameliorates oxidative stress-induced inflammation, apoptosis, and heart failure in isoproterenol-challenged Wistar rats. Iran J Basic Med Sci.

[3] Liao L, Song D, Shi B, Chen M, Wu L, Xu J, Dong F (2022). Inhibition of CCR8 attenuates Ang Ⅱ-induced vascular smooth muscle cell injury by suppressing the MAPK/NF-κB pathway. Iran J Basic Med Sci.

[4] Parmar MP, Kaur M, Bhavanam S, Mulaka GS, Ishfaq L, Vempati R (2023). A systematic review of the effects of smoking on the cardiovascular system and general health. Cureus.

[5] Benowitz NL, Liakoni E (2022). Tobacco use disorder and cardiovascular health. Addiction.

[6] Kopp W (2022). Pathogenesis of (smoking-related) non-communicable diseases–evidence for a common underlying pathophysiological pattern. Front Physiol.

[7] Poznyak AV, Sadykhov NK, Kartuesov AG, Borisov EE, Melnichenko AA, Grechko AV (2022). Hypertension as a risk factor for atherosclerosis: Cardiovascular risk assessment. Front Cardiovasc Med.

[8] Hertiš Petek T, Marčun Varda N (2024). Childhood cardiovascular health, obesity, and some related disorders: Insights into chronic inflammation and oxidative stress. Int J Mol Sci.

[9] Bludau DC, Pabst A, Bleck F, Weyerer S, Maier W, Gensichen J (2025). Overweight, obesity, and depression in multimorbid older adults: Prevalence, diagnostic agreement, and associated factors in primary care results from a multicenter observational study. Nutrients.

[10] Oral O, Maqbool M, Thapa P, Tatlibal P, Enser M (2025). The potential impact of weight control management on metabolic health during healthy aging. Global Transl Med.

[11] Sui X, Kokkinos P, Faselis C, Samuel IB, Pittaras A, Gollie J (2025). Cardiorespiratory fitness and mortality in patients with chronic kidney disease: A prospective cohort study. Mayo Clin Proc.

[12] Chen F, Chen R, Yang L, Shen B, Wang Y, Gao Y (2025). Magnesium-assisted hydrogen improves isoproterenol-induced heart failure. Med Gas Res.

[13] Liu J, Li W, Jiao R, Liu Z, Zhang T, Chai D (2025). Miglustat ameliorates isoproterenol-induced cardiac fibrosis via targeting UGCG. Mol Med.

[14] Goumtsa AF, Nguelefack-Mbuyo EP, Nokam F, Koho CW, Dial CM, Nguelefack TB (2025). Antihypertrophic effects of the seed ethanolic extract of Aframomum pruinosum Gagnep (Zingiberaceae) against isoproterenol-induced cardiac hypertrophy in male Wistar rat. Toxicol Rep.

[15] Arandhara A, Saha D, Das BK (2025). Evaluation of the cardioprotective potential of hydroethanolic extract of Koenigia polystachya L (Qiú xù liǎo) leaves against isoproterenol-induced myocardial infarction in rats. Pharmacol Res TCM.

[16] Zhao XL, Cao ZJ, Li KD, Tang F, Xu LY, Zhang JN (2025). Gallic acid: A dietary metabolite’s therapeutic potential in the management of atherosclerotic cardiovascular disease. Front Pharmacol.

[17] Zhuang P, Liu X, Li Y, Ao Y, Wu Y, Ye H (2025). A global analysis of dairy consumption and incident cardiovascular disease. Nat Commun.

[18] Cámara M, Fernández-Ruiz V, Sánchez-Mata MC, Cámara RM, Domínguez L, Sesso HD (2022). Scientific evidence of the beneficial effects of tomato products on cardiovascular disease and platelet aggregation. Front Nutr.

[19] Khan UM, Sevindik M, Zarrabi A, Nami M, Ozdemir B, Kaplan DN (2021). Lycopene: Food sources, biological activities, and human health benefits. Oxid Med Cell Longev.

[20] Przybylska S, Tokarczyk G (2022). Lycopene in the prevention of cardiovascular diseases. Int J Mol Sci.

[21] Agrawal M, Singhal M, Gupta R, Bhargava S, Bisht D, Arya RK (2024 ). Nutraceutical Herbs in Cardiovascular Diseases. Herbals as Nutraceuticals.

[22] Zamani M, Behmanesh Nia F, Ghaedi K, Mohammadpour S, Amirani N, Goudarzi K (2023). The effects of lycopene and tomato consumption on cardiovascular risk factors in adults: A grade assessment systematic review and meta-analysis. Curr Pharm Des.

[23] Arballo J, Amengual J, Erdman Jr JW (2021). Lycopene: A critical review of digestion, absorption, metabolism, and excretion. Antioxidants.

[24] Duceac IA, Vereștiuc L, Coroaba A, Arotăriței D, Coseri S (2021). All-polysaccharide hydrogels for drug delivery applications: Tunable chitosan beads surfaces via physical or chemical interactions, using oxidized pullulan. Int J Biol Macromol.

[25] Mahadevan G, Brahma RK, Kini RM, Valiyaveettil S (2023). Purification of intramineral peptides from cuttlebones and in vitro activity in CaCO3 biomineralization. Langmuir.

[26] Dardi P, Perazza LR, Couto GK, Campos GP, Capettini LD, Rossoni LV (2021). Vena cava presents endothelial dysfunction prior to thoracic aorta in heart failure: The pivotal role of nNOS uncoupling/oxidative stress. Clin Sci.

[27] Albrahim T (2022). Lycopene modulates oxidative stress and inflammation in hypercholesterolemic rats. Pharmaceuticals.

[28] Deng J, Jiang Y, Chen ZB, Rhee JW, Deng Y, Wang ZV (2023). Mitochondrial dysfunction in cardiac arrhythmias. Cells.

[29] Wang J, Fu J, Chen D (2021). Study on the protective effect of Lycopene on ischemia-reperfusion myocardium through Inhibiting the opening of mitochondrial MPTP and the activation of apoptotic pathway. Food Sci Technol.

[30] Cobb AM, Yusoff S, Hayward R, Ahmad S, Sun M, Verhulst A (2021). Runx2 (runt-related transcription factor 2) links the DNA damage response to osteogenic reprogramming and apoptosis of vascular smooth muscle cells. Arterioscler Thromb Vasc Biol.

[31] Nusier M, Elimban V, Prasad J, Shah AK, Dhalla NS (2023). Regulatory role of some protein kinases in signal transduction pathways in heart health and disease. Scr Med.

[32] Miao R, Wang L, Chen Z, Ge S, Li L, Zhang K (2022). Advances in the study of nicotinamide adenine dinucleotide phosphate oxidase in myocardial remodeling. Front Cardiovasc Med.

[33] Ahn H, Cho Y, Yun GT, Jung KB, Jeong W, Kim Y (2023). Hierarchical topography with tunable micro‐and nanoarchitectonics for highly enhanced cardiomyocyte maturation via multi‐scale mechanotransduction. Adv Healthc Mater.

[34] Wang H, Lin Y, Liu Q, Zhou A, Bian H, Zhang W (2023). Antioxidant, anticancer activity and molecular docking study of lycopene with different ratios of Z-isomers. Curr Res Food Sci.

[35] Cao G, Wang H, Wang X, Jia K, Yang H, Wang E (2024). Protective effects of Lycopene on growth performance, intestinal morphology, cecal microflora, and serum metabolome in heat-stressed broilers. Poultry Sci.

[36] Long Y, Paengkoum S, Lu S, Niu X, Thongpea S, Taethaisong N (2024). Physicochemical properties, mechanism of action of lycopene and its application in poultry and ruminant production. Front Vet Sci.

[37] Gao X, Lin B, Chen C, Fang Z, Yang J, Wu S (2024). Lycopene from tomatoes and tomato products exerts renoprotective effects by ameliorating oxidative stress, apoptosis, pyroptosis, fibrosis, and inflammatory injury in calcium oxalate nephrolithiasis: The underlying mechanisms. Food Funct.

[38] Li M, Tang S, Peng X, Sharma G, Yin S, Hao Z (2024). Lycopene as a therapeutic agent against aflatoxin b1-related toxicity: Mechanistic insights and future directions. Antioxidants.

